# Exploratory study on the role of emotion regulation in perceived valence, humour, and beneficial use of depressive internet memes in depression

**DOI:** 10.1038/s41598-020-57953-4

**Published:** 2020-01-21

**Authors:** Umair Akram, Jennifer Drabble, Glhenda Cau, Frayer Hershaw, Ashileen Rajenthran, Mollie Lowe, Carissa Trommelen, Jason G. Ellis

**Affiliations:** 1 Department of Psychology, Sociology and Politics, Sheffield Hallam University, Sheffield, UK; 2 Nuffield Department of Clinical Neurosciences, University of Oxford, Oxford, UK; 3 Department of Psychology, Faculty of Health & Life Sciences, Northumbria University, Northumbria, UK

**Keywords:** Human behaviour, Risk factors

## Abstract

This study examined whether individuals experiencing significant depressive symptoms would differ from non-depressed controls in their interpretation of internet memes related to depression, whilst incorporating the mediating role of emotion regulation difficulty. Forty-three individuals presenting clinically significant depressive symptoms (indicating ≥15 on the PHQ-9) and 56 non-depressed controls (indicating ≤4) rated the emotional valance, humour, relatability, shareability, and mood improving potential of 32 depressive and control (depicting general neutral or positive social commentaries) internet memes. Measures of depression and emotion dysregulation were also completed. The perception of humour, relatability, shareability and mood improving potential of depressive, but not control, memes were all greater amongst individuals with symptoms of depression relative to controls. However, these differences were mediated by deficits in the ability to deploy adaptive emotion regulation strategies. Despite their negative orientation, internet memes related to depression may be beneficial for individuals experiencing consistent symptoms. Specifically, by potentially facilitating: a humorous take on a negative experience and situation; the perception of peer-support through affiliation with others experiencing similar symptoms; and adaptive emotion regulation strategies amongst those with deficits in the ability to deploy such strategies.

## Introduction

Major Depressive Disorder is predominately characterized by disordered affect (i.e. sustained negative affect, absence of positive affect)^1^ and is associated with marked cognitive (e.g. impaired memory and executive control)^2,3^ and behavioural (e.g. social and inter-personal function) deficits, and difficulties in the adequate regulation of emotions. Symptoms of depression are highly prevalent, affecting up to 27% of the general population according to recent meta analytic data^4^.

A number of cognitive models have been advanced to explain the mechanisms underlying the development and maintenance of depression^5^. Emphasized in these models is the notion that depressed individuals, and those at risk for depression, display cognitive biases in several aspects of information processing including attention, interpretation and memory^6–8^. It is speculated that these biases are more than epiphenomenal in nature, serving a causal role in the development and maintenance of the disorder^9^. Indeed, it is well established that depressed individuals often display an elevated perception of negative stimuli and interpret ambiguous neutral information in a disorder congruent manner^10–15^. With that in mind, the valence in which such information/stimuli are interpreted (i.e. positive vs. negative) governs the strength and direction of an emotional response^16^. In turn, interpretative biases may be mediated by an individual’s ability to employ effective emotion-regulation strategies when required.

Another key feature associated with the experience of depression is impairment in social functioning^17^. Here, any perceived reward value related to social interaction appears to be decreased in depressed individuals^18^, which may partially explain the reduction of social behaviour in this population^19^. As mentioned, depressed individuals display a tendency to interpret ambiguous information in a disorder congruent manner^8^. Relatedly, similar deficits in the perception of emotional stimuli are evidenced, whereby a mood congruent bias emerges in the presence of affective stimuli (i.e. emotional faces and prosody)^20–22^. In particular, when compared with healthy controls, individuals experiencing symptoms of depression display impairments in the discrimination of emotionally neutral, happy, and sad faces^21–23^. However, examination of social cognition deficits in depression remain limited to traditional response time and emotion perception tasks largely using words or emotional faces as stimuli^24^. To that end, using a novel humour-processing task, Uekermann and colleagues^24^ examined cognitive and affective humour processing in depression. Specifically, depressed patients and healthy controls were compared in valance ratings and the ability to accurately determine punchlines for presented jokes. Deficits in social cognition emerged amongst those with depression in affective (i.e. blunted humour ratings) and cognitive (i.e. impaired accuracy) aspects of humour processing.

The experience of humour is considered vital in maintaining physical and psychological wellbeing^25^. However, supporting evidence remains limited to specific populations (e.g. individuals experiencing experimentally induced stress) and style of humour (e.g. positive vs negative) examined^26^. In healthy subjects, positive styles (affiliative and self-enhancing) are more effective in down-regulating negative and up-regulating positive emotion when compared to negatively oriented humour (aggressive and self-defeating)^27^. Therefore, positive humour may function as an effective form of emotion regulation whereby the accompanying positive emotion serves to facilitate the reappraisal of negative emotions^28^. That said, research has yet to examine whether depressed individuals respond to positive humour in the same way. Tentatively, Perchtold and colleagues^29^ evidence symptoms of depression to be associated with individual differences in the strategic approach taken when using humour to facilitate cognitive reappraisal. Here, those indicating symptoms of depression favoured the comparative approach, where situational threat is compared with another more threatening event (i.e. it could be worse). In contrast, non-depressed individuals reported preferential attention for positive situational factors, which remain detached from threat (e.g. appreciating the surroundings or accomplishments of the day).

Few studies use stimuli directly related to the experience of depressive symptoms^26^. Rather, most studies examining humour perception in depression rely on self-report questionnaire measures, whereas experimental studies generally use positively valanced stimuli (i.e. amusing photographs and films)^26^. Depressed individuals potentially differ in their conceptualisation of positive and negative humour compared non-depressed people. In particular, negatively oriented humour may appeal to this population when considering the relatability and salience to the experience of depression. If true, negative humour related to the experience of depression may also serve to regulate emotion in a comparative manner.

Despite reduced face-to-face social interaction^18^, perceived social support via interaction with others on the internet appears beneficial in reducing symptoms of depression in a sample of US college students^30^. With that in mind, the experience of depression has been frequently linked to prolonged internet use^31–34^ and anecdotally, the frequent observation and sharing of memes. Internet memes are an element of a culture or system of behaviour (e.g. an image with a caption) that are widely distributed by groups of people with shared characteristics of experiences through electronic means^35^. Typically, memes depict humorous social commentaries which are contextually relevant to a particular demographic of individuals^36^. A number of social media sites and forums host various pages dedicated to the sharing of memes specifically related to the proximal experience of depression, often termed *depressive memes*. Whilst highly prevalent, research has yet to examine how symptoms of depression may influence, or be influenced by, the interpretation of affective internet memes related to the experience of depression (i.e. depressive memes). Engaging with media (i.e. television, music, internet) is known to regulate general mood state (i.e. regardless of valance). However, emotion regulation deficits of limited awareness and coping strategies predict increased media use in the occurrence of a negative mood state^37^. When used adaptively, emotion regulation strategies (i.e. cognitive reappraisal, distraction) can increase: expression of positive emotion; interpersonal functioning; and psychological wellbeing^38^. In the current context, depressive memes could diminish the meaning of particular events (i.e. perspective placement) while concurrently allowing one to make light of a negative experience (i.e. positive appraisal).

The goal of the present research was to examine whether individuals experiencing symptoms of depression interpret depressive memes differently as compared to non-depressed controls. More specifically, we examined group differences in the perception of emotional valance, humour, relatability and shareability of depressive and control (depicting general neutral or positive social commentaries) internet memes. In addition, participants assessed the mood improving potential of each meme. Finally, difficulties in emotion regulation mediate the relationship between depression with humour perception and cognitive biases of information processing^29^. As such, we examined the extent to which deficits in emotion regulation mediated any confirmed perceptual differences. As the first study to examine how symptoms of depression may influence the interpretation of depressive memes, we consider this to be an exploratory investigation with no a-priori hypotheses.

## Method

### Participants

A cross-sectional online questionnaire-based survey was implemented comprising of questions designed to examine emotion dysregulation, symptoms of depression and the perception of depressive and control internet memes. The survey was advertised to members of the general population through social media, ‘call for participants’ (website), and students from Sheffield Hallam University through the institutions course participation scheme. This resulted in a sample of N = 200 individuals who began the survey, and 154 respondents (mean age = 23.64 ± 10.12, range 18–56, 74% female) providing complete data.

### Materials

#### The patient health questionnaire

The Patient Health Questionnaire (PHQ-9)^39^ assessed depressive symptoms. Nine items capture core depressive symptoms as outlined in the DSM-IV/DSM-5. Items are scored on a 4-point likert scale (0 = not at all, 1 = several days; 2 = more than half of the days; 3 = nearly all days). The summation of items provides a total score between 0–27, where higher scores indicate greater levels of depressive symptoms. Specifically, 0–4 indicates none-minimal severity, 5–9 mild, 10–14 moderate, 15–19 moderately severe and 20–27 severe depression. Internal consistency (Cronbach’s α) of the scale in the present study was 0·92.

#### Emotion dysregulation

The Short Form Difficulties in Emotion Regulation Scale (DERS-SF)^40^ assessed individuals’ ability to adequately regulate emotions. Six subscales examine: *nonacceptance* (nonacceptance of emotional states); *goals* (difficulties engaging in goal directed behaviour in the context of emotional distress); *impulse* (difficulty controlling behaviours when upset); *awareness* (lack of emotional awareness); *strategies* (limited access to adaptive emotion regulation skills); and *clarity* (lack of emotional clarity). Each subscale is comprised of three items scored on a 5-point likert scale ranging from 1 (almost never) to 5 (almost always). Mean scores are created for each subscale, with higher scores indicating greater difficulty in emotion regulation. Internal consistency of subscales in the present study: Nonacceptance, 0·90; Goals, 0·92; Impulse, 0·93; Awareness, 0·82; Strategies, 0·90; and Clarity, 0·83.

#### Pictorial stimuli

In the absence of an existing picture set comprised of memes relating to depression, a new set was developed and validated within the context of this study. Pictorial memes relating to depression were obtained from an online google image search using keywords such as ‘depression’, ‘depressive’, ‘humorous’, ‘everyday’, ‘social’ and ‘memes’. Consequently, we identified fifty-two memes each comprised of an image paired with a short amount of text. Of these, 26 contained affective content relating to the experience of depression (i.e. depressive memes). In contrast, the remaining reflected generally humorous social commentaries (control memes). The final stimuli set included in the survey was agreed by all eight members of the research team (three post-doctoral psychologists and five clinical and cognitive neuroscience MSc candidates) following thorough consideration based on a number of factors. Specifically, for depressive memes: their likelihood of inducing a degree of valance and arousal; and presence of affective content relevant to the experience of depression (e.g. death, suicide, isolation, hopelessness, hypersomnia) as outlined in the DSM-5 criteria for Major Depressive Disorder (American Psychiatric Association, 2013). For the control memes: no relation to the experience of depression; humorous content; and relatability to everyday experiences and social situations. Following further review of the stimuli conducted by two post-doctoral psychologists independent of the research team, the final images were standardised for presentation size 640 × 640 px.

### Procedure

Ethical approval was granted by the the Sheffield Hallam University Research Ethics Committee. This experiment was conducted in accordance with the Declaration of Helsinki, and all participants gave their written informed consent before participation.

Participants completed the online questionnaire, in which they were presented the series of 56 pictorial memes in randomized order. Using a 5-point likert scale ranging from strongly disagree (=1) to agree (=5), participants reported the extent to which each meme was considered to be: positive; relatable; funny; something they would share with other people; and something that would make a person with depression feel good. In addition, participants categorically indicated whether each meme was perceived to be more related to anxiety, depression, or neither. Following the pictorial meme ratings, the PHQ-9 and DERS-SF was administered. Once complete, participants were debriefed about the nature of the study. Students who requested course credit were remunerated on completion.

### Statistical analyses

#### Stimuli validation

Preliminary analysis confirmed the extent each pictorial meme accurately depicted its corresponding group (i.e. depressive or control), with those deemed unrepresentative consequently discarded from final analysis. First, the percentage of agreement in corresponding categorisation was examined. This was based on participants’ responses when asked to indicate: whether each meme they perceived to be more related to anxiety, depression, or neither. Following, based on the extent memes were considered positive (strongly disagree = 1 to agree = 5) mean valance ratings were calculated. Depressive memes with a mean valance above 2.5 were discarded. Similarly, control memes below 3.0 were discarded.

**Figure 1:**
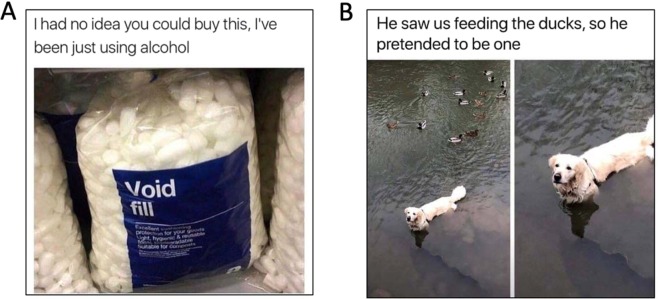
(**A**) Example of depressive meme; (**B**) example of control meme. All images used were gathered from the public domain each marked with either the Public Domain Mark 1.0 or CC0 1.0 Universal licence. No alterations were made. Title & Artist: Unknown. Image Source: Snappygoat.

Sixteen depressive memes with an acceptable level of categorisation agreement (all > 77%) were yielded, where depression was indicated over anxiety or neither. For these, valance ratings remained below 2.5 for all 16 thus were retained (see Fig. 1b for an example). Nineteen control memes with acceptable agreement (all > 77%) were also yielded, where neither was indicated over anxiety or depression. A further three were discarded based on mean valance ratings below 3, leaving 16 control memes for analysis (see Fig. 1a for an example).

The mean ratings for the final set of 16 depressive and 16 control memes were calculated for the whole sample. Paired samples t-tests confirmed that control memes were rated as significantly more positive (3.53 ± 0.57) relative to the depressive memes (2.04 ± 0.53: *t*(153) = 23.37, p = 0.001). Examination of internal consistency (Cronbach’s α) for depressive memes yielded a high degree of consistency: α = 0·92. Likewise, this was also apparent for control memes: α = 89. Therefore, the 16 final memes reliably depict their corresponding group (i.e. depressive or control) with a degree of confidence.

#### Statistical analysis

##### Participant Grouping

Participants were first grouped based on the severity of reported depression symptoms. Using the PHQ-9, individuals with a score of: ≤4 (Mean = 2.02 ± 1.29; Range = 0–4) were placed into the control group (n = 56: mean age = 25.16 ± 9.30; 71% female); and ≥15 (Mean = 19.86 ± 3.34; Range = 15–27) into the depression symptoms group (n = 43: mean age = 22.42 ± 7.44; 74% female). Data from N = 55 individuals who did not meet the criteria for either group were discarded at this point. Following, mean perception ratings for depressive and control memes were calculated for the whole sample and each individual group. This was conducted for each parameter assessed. Specifically: a) valance, as assessed by asking the extent to which each meme was considered to be positive (i.e. valance); b) humor (or funniness); c) relatability; d) shareability; and e) something that would make a person with depression feel good (DEPFEEL).

##### Analysis

A series of 2 (group: control vs. depression symptoms) × 2 (meme type: depressive vs. control) mixed measures ANOVA analysis were employed, with rating scores for each parameter as dependent variables. This was conducted to assess the main effects of group and meme type, as well as the group x meme type interaction. Moreover, simple effects analyses were performed to determine any significant interactions as appropriate. This was followed by a series of sequential logistic regression analyses to determine whether group (control vs. depression symptoms) differences in the perception of depressive memes were mediated by emotion dysregulation. For example, valence ratings (step 1), and emotional dysregulation subscales (nonacceptance, goals, impulse, awareness, strategies, clarity: step 2) were entered as predictor variables. Significance was considered at the P < 0.05 level.

## Results

Mean scores on the PHQ-9 and DERS-SF, as well as meme ratings for each group, are displayed in Table 1.

**Table 1:** Ratings of memes for the control and depression symptoms groups whilst observing depressive and control memes (means ± standard deviation).

	Depressive Memes		*t*	P	Cohens’ *d*
	Depression Symptoms (n = 43)	Control Group (n = 56)			
**Ratings:**					
Valence	2.14 ± 0.70	1.90 ± 0.66	−1.69	0.093	0.35
Humor	3.81 ± 0.99	2.78 ± 0.91	−5.73	0.001**	1.08
Relatability	3.08 ± 0.81	1.94 ± 0.75	−10.80	0.001**	1.46
Shareability	3.17 ± 1.26	2.01 ± 0.86	−5.44	0.001**	1.08
DEPFEEL	2.79 ± 0.98	2.19 ± 1.02	−2.91	0.004*	0.60
	**Control Memes**				
Valence	3.56 ± 0.63	3.44 ± 0.56	−0.91	0.364	0.20
Humor	3.57 ± 0.87	3.67 ± 0.62	0.63	0.532	0.16
Relatability	3.08 ± 0.81	2.89 ± 0.63	−1.30	0.197	0.26
Shareability	2.82 ± 1.06	2.82 ± 0.94	−0.03	0.980	0.00
DEPFEEL	3.20 ± 0.60	3.20 ± 0.50	−0.00	0.999	0.00
PHQ-9	19.86 ± 3.34	2.02 ± 1.29			
**DERS-SF:**					
Nonacceptance	3.47 ± 1.28	1.77 ± 0.83	−7.96	0.001**	
Goals	4.09 ± 1.10	2.47 ± 0.95	−7.86	0.001**	
Impulse	2.99 ± 1.36	1.55 ± 0.79	−6.59	0.001**	
Awareness	2.87 ± 1.07	2.32 ± 0.98	−2.67	0.009*	
Strategies	3.68 ± 1.02	1.53 ± 0.61	−13.12	0.001**	
Clarity	2.94 ± 1.06	1.79 ± 0.83	−6.05	0.001**	

Note: DEPFEEL, ratings of the extent to which memes would make someone with depression feel good; PHQ-9, Patient Health Questionnaire Depression Scale; DERS-SF, Difficulties in Emotion Regulation Scale Short Form.

*Sig at < 0.01, ** < 0.001.

### Valance

The results revealed a significant main effect of meme type (F(1,96) = 310.17, p = 0.001) on ratings of valence. However, no main effects of group (F(1,96) = 2.49, p = 0.118) or group x meme type interactions (F(1,96) = 0.91, p = 0.342) were determined.

### Humour

The results demonstrated significant main effects of group (F(1,97) = 10.94, p = 0.001) and meme-type (F(1,97) = 10.91, p = 0.001) on humour ratings. Moreover, a significant group x meme type interaction demonstrated that, compared to control participants (2.78 ± 0.91), individuals experiencing symptoms of depression (3.81 ± 0.99) rated the depressive memes as significantly more humorous, F(1,97) = 32.96, p = 0.001. Individual analysis of humour ratings for each meme type revealed a significant difference between individuals experiencing symptoms of depression (3.81 ± 0.99) and control participants (2.78 ± 0.91) for depressive memes only (*t*(97) = −5.37, p = 0.001). No group differences were observed for neutral memes (*t*(97) = −0.63, p = 0.53).

### Relatability

The results revealed a significant main effect of group (F(1,97) = 59.95, p = 0.001) on ratings of relatability. Moreover, a significant group x meme type interaction demonstrated that, compared to control participants (1.94 ± 0.75), the depression symptoms group (3.80 ± 0.81) rated depressive memes as significantly more relatable, F(1,97) = 89.63, p = 0.001. No main effect of meme type (F(1,97) = 1.71, p = 0.194) was observed. Individual analysis of relatability ratings for each meme type revealed a significant difference between individuals experiencing symptoms of depression (3.08 ± 0.81) and control participants (1.94 ± 0.75) for depressive memes only (*t*(97) = −10.80, p = 0.001). No group differences were observed for neutral memes (*t*(97) = −1.30, p = 0.20).

### Shareability

The results revealed significant main effects of group (F(1,96) = 9.75, p = 0.002) and meme-type (F(1,96) = 6.28, p = 0.014) on the likelihood of sharing memes with other people. Moreover, a significant group x meme type interaction demonstrated that, compared to control participants (2.01 ± 0.86), the depression symptoms group (3.17 ± 1.26) rated depressive memes as significantly more sharable, F(1,96) = 40.00, p = 0.001. Individual analysis of shareability ratings for each meme type revealed a significant difference between individuals experiencing symptoms of depression (3.17 ± 1.26) and control participants (2.01 ± 0.86) for depressive memes only (*t*(96) = −5.44, p = 0.001). No group differences were observed for neutral memes (*t*(96) = −0.03, p = 0.98).

### DEPFEEL

The results revealed significant main effects of group (F(1,97) = 6.32, p = 0.011) and meme-type, F(1,97) = 38.88, p = 0.001. Moreover, a significant group x meme type interaction demonstrated that, compared to control participants (2.19 ± 1.02), the depression symptoms group (2.79 ± 0.98) rated depressive memes as significantly more likely to improve the mood of someone with depression, F(1,97) = 6.76, p = 0.011. Individual analysis of ratings for each meme type revealed a significant difference between individuals experiencing symptoms of depression (2.79 ± 0.98) and control participants (2.19 ± 1.02) for depressive memes only (*t*(97) = −2.91, p = 0.004). No group differences were observed for neutral memes (*t*(97) = −0.00, p = 0.99).

### Mediating role of emotion regulation

Sequential logistic regression analysis demonstrated that the extent of humour ratings whilst observing depressive memes (step 1: 23% variance explained; see Table 2a) significantly predicted group status control vs. depression symptoms. However, after accounting for subscales of emotional dysregulation (nonacceptance, goals, impulse, awareness, strategies, clarity), strategies remained the only significant predictor of group stats in the following step (step 2: 60% variance). Interestingly, this pattern of results was mirrored for individual analysis of relatability, shareability and DEPFEEL, whereby emotion regulation strategies remained the only significant predictor of group status (see Table 2b–d respectively).

**Table 2:** Sequential logistic regression analyses with group status (control vs. depression symptoms) as the dependant variable; meme ratings and DERS-SF subscales as predictors.

*Predictors*	R^2^	β	Wald	Sig.
[A]				
Step 1	0.23			
Humour		1.13	18.52	0.001**
Step 2	0.60			
Humour		0.58	1.67	0.196
Awareness		1.05	3.81	0.051
Clarity		−0.18	0.11	0.746
Nonacceptance		0.50	1.19	0.275
Impulse		0.00	0.00	0.993
Goals		0.31	0.35	0.555
Strategies		2.55	10.35	0.001**
[B]				
Step 1	0.50			
Relatable		2.14	26.68	0.001**
Step 2	0.63			
Humour		1.61	6.10	0.013*
Relatable		1.31	3.19	0.074
Clarity		−0.16	0.06	0.801
Nonacceptance		0.44	0.68	0.409
Impulse		−0.23	0.22	0.643
Goals		0.28	0.25	0.615
Strategies		2.37	7.44	0.006*
[C]				
Step 1	0.22			
Shareable		0.97	18.92	0.001**
Step 2	0.60			
Shareable		0.58	1.56	0.211
Awareness		0.92	3.40	0.065
Clarity		−0.12	0.00	0.982
Nonacceptance		0.33	0.53	0.468
Impulse		0.12	0.08	0.776
Goals		0.41	0.59	0.443
Strategies		2.36	9.01	0.003*
[D]				
Step 1	0.08			
DEPFEEL		0.58	7.54	0.006*
Step 2	0.60			
DEPFEEL		0.60	1.77	0.183
Awareness		1.01	3.76	0.052
Clarity		−0.10	0.03	0.859
Nonacceptance		0.55	1.35	0.245
Impulse		−0.62	0.02	0.889
Goals		0.32	0.38	0.540
Strategies		2.64	11.14	0.001

*Note:* DEPFEEL, ratings of the extent to which memes would make someone with depression feel good; PHQ-9, Patient Health Questionnaire Depression Scale; DERS-SF, Difficulties in Emotion Regulation Scale Short Form.; R²: Cox & Snell R Square.

*Sig at < 0.01, ** < 0.001.

Therefore, compared to non-depressed controls, individuals experiencing symptoms of depression perceive depressive memes as more humorous, relatable, and shareable with the potential of improving the mood of a person with depression. However, these differences in interpretation appear to be mediated by limitations in the ability to deploy adaptive emotion regulation strategies.

## Discussion

This study examined whether individuals experiencing significant depressive symptoms would differ from non-depressed controls in their interpretation of internet memes related to depression, whilst incorporating the mediating role of emotion regulation difficulty. With the exception of valence ratings, groups differed in their interpretation of depressive memes. More specifically, the perception of humour, relatability, shareability and mood improving potential of depressive, but not control, memes were all greater amongst individuals with symptoms of depression relative to non-depressed controls. However, these differences are mediated by deficits in the ability to deploy adaptive emotion regulation strategies. To our knowledge, this is the first report to show that individuals experiencing depressive symptoms differ with respect to their interpretation of affective internet memes.

The experience of depression is associated with alterations in cognitive processing, whereby the perception of emotionally salient and ambiguous stimuli are frequently perceived in a disorder congruent manner^10–15^. Despite the affective nature and content of depressive memes currently used (e.g. death, suicide, isolation, hopelessness), groups failed to differ in valance judgments. Whilst no evidence of disorder consistent information was evidenced, this may be explained by low valance ratings (i.e. as less positive) provided by both groups in regard to depressive memes.

Considering the salience of depressive memes for those currently experiencing symptoms of depression, greater relatability ratings observed amongst this population is perhaps not surprising. Nevertheless, the most novel outcomes of this study were: the disproportionate tendency for individuals presenting symptoms of depression to perceive depressive memes as significantly more humorous and sharable with the potential to improve the mood of others with depression; and the role of emotion regulation difficulty as a mediating factor.

Positively oriented humour has previously been shown to be beneficial in reducing emotional distress and correcting the regulation of emotion in non-depressed individuals^28,30,31,41^. In contrast, our findings highlight the potential benefits of a more negative style of humour, at least for those experiencing symptoms of depression. That said, whilst depressive memes typically embody a negative style of humour (i.e. aggressive and self-defeating), their affiliative nature to those experiencing depression may be considered contextually positive. Indeed, definitionally, memes are humorous social commentaries which are contextually relevant to a particular demographic of individuals^32^. Perceived social support through online interaction appears beneficial in reducing symptoms of depression^42^. Memes visualise the experience and encumbering nature of depressive symptoms, which for many may be difficult to verbalise. Therefore, by sharing and observing depressive memes, depressed individuals may theoretically form social and emotional bonds with others which may be perceived as socially supportive.

Emotion regulation relates to how emotions are controlled, experienced and expressed over a brief time period^43^. Here, our data suggests perceptual differences in the observation of depressive memes were mediated by deficits in the ability to deploy adaptive emotion regulation strategies (e.g. positive refocusing). In the context of social media use, emotion regulation is considered vital in upregulating positive (e.g. viewing enjoyable posts and pictures) and downregulating negative emotions (e.g. reading posts related to coping with depression)^44^. Emotion regulation strategies often diminish with depression^42^. With that in mind, an increased disposition for maladaptive strategies (e.g. self-directed hostility, rumination) could increase depressive meme use in this population as a means to express negative emotion. In contrast, depressive memes used in an adaptive way might diminish the meaning of certain events (i.e. perspective placement) while allowing the individual to make light of a negative experience (i.e. positive appraisal). Certainly, given their wide availability, depressive memes may even lead an individual to recognise that they are not alone in the experience of their symptoms. However, when asked about the mood improving potential of depressive memes, those experiencing depressive symptoms likely self-reflect in their response (i.e. *how would this make me feel?, other people in my position should therefore feel the same*) whereas non depressed controls, potentially, are more likely to reference another (i.e. *how would this make my friend with depression feel?*).

Several limitations of the current study should be noted. The current sample consisted primarily of female participants, and as such the present findings may not be fully generalizable to males. Moreover, whilst the present study used a comprehensive assessment to address depressive symptoms amongst the general population from the perspective of diagnostic criteria, the current outcomes cannot be extrapolated to individuals meeting diagnostic criteria for major depressive disorder. To that end, a replication of the current study amongst a sample meeting diagnostic criteria would be beneficial.

In sum, the perception of humour, relatability, shareability and mood improving potential of depressive memes were all greater amongst individuals with symptoms of depression relative to controls. However, these differences were mediated by deficits in the ability to deploy adaptive emotion regulation strategies. Despite the negative orientation, engagement with internet memes related to depression may be beneficial for individuals experiencing consistent symptoms. Specially, by potentially facilitating: a humorous take on a negative experience and situation; the perception of peer-support through affiliation with others experiencing similar symptoms; adaptive emotion regulation strategies amongst those with deficits in the ability to deploy such strategies. More research is now required to further understand how individuals with depression engage with depressive memes, which ultimately may allow for potential therapeutic use of depressive memes to be established.
